# The Adsorptive Removal of Fluoride from Aqueous Solution by Modified Sludge: Optimization Using Response Surface Methodology

**DOI:** 10.3390/ijerph15040826

**Published:** 2018-04-23

**Authors:** Ying Li, Shengke Yang, Qianli Jiang, Jie Fang, Wenke Wang, Yanhua Wang

**Affiliations:** 1 Key Laboratory of Subsurface Hydrology and Ecological Effects in Arid Region, Ministry of Education, Chang’an University, Xi’an 710054, China; 15291192057@163.com (Y.L.); jiangqianli123@sina.cn (Q.J.); 18792975240@163.com (J.F.); wenkew@chd.edu.cn (W.W.); yhwang930@foxmail.com (Y.H.); 2 Xi’an Center of Geological Survey, China Geological Survey, Xi’an 710054, China; 3 School of Geography and Tourism, Shaanxi Normal University, Xi’an 710054, China

**Keywords:** fluoride, response surface methodology, water supply plant sludge, adsorption

## Abstract

The sludge from the water supply plant was investigated to remove fluoride ions from the water. To improve the adsorption ability, the original sludge sample was treated with fuel oxidation, pyrolysis, hydrochloric acid, and sulphuric acid methods, and hydrochloric acid treatment improved the adsorption capacity of the sludge on the fluoride in water significantly, with a maximum adsorption capacity to 140 mg/kg. The adsorption experimental data was the well fitted pseudo-first-order model and the Langmuir isotherms model. SEM images and XRD patterns of the adsorbent were recorded to get a better insight into the adsorption process. The effect of three variables, hydrochloric acid treated sludge (HWS) dose, pH, and initial fluoride concentration were studied using a Box-Behnken statistical experimental design. The model of the adsorption and optimum conditions was investigated using the response surface methodology. The optimum removal efficiency of fluoride can reach 81.153% under the optimum condition: HWS dose of 14.10 g/L and pH value at 6.12. The effect of co-existing anions and the removal efficiency from the water were also studied. The results suggest that sludge from the water supply plant can be reused as a coagulant for the removal of fluoride from poor quality water.

## 1. Introduction

As a natural element, fluoride is universally present in varied water bodies, and it is considered beneficial up to 0.7 mg/L but detrimental if it exceeds 1.5 mg/L, which is the limit recommended by the World Health Organization (2004). Fluoride pollution has been a global environmental concern for decades and has caused great concerns due to its widespread nature and threat to human health. Human exposure to over 1.5 mg/L of fluoride often causes a lot of health problems through drinking water, such as brain damage, dental fluorosis and skeletal fluorosis, and thyroid disorder [1]. Fluoride-polluted surface water and groundwater is ubiquitous, especially in India, Africa, and the southwest of China. Fluoride has been found in groundwater in many parts of the globe, and it is present in at least 25 countries [2,3].

In the past decades, various techniques have been developed to remove excessive fluoride ions from river water, including coagulation-precipitation [4], membrane-based process [5], ion exchange [6], and adsorption [7,8]. In generally, adsorption has been regarded as one of the most important and widely used approaches in the defluoridation of wastewater [9]. Many adsorbents have been used for defluoridation, including activated alumina [10], carbonaceous materials [11], activated clay [12], rare earth oxides [13], titanium rich bauxite [14], zeolites [15], and modified natural polymers [16]. Recently, alum sludge has been derived from the by-products of the alum plant, which has been used in environment-related fields to reduce soluble phosphate and nitrogen in soil runoff, land-applied organic waste, and adsorption of F^−^ in aqueous systems [16,17]. The adsorption characteristics of alum sludge for fluoride varies according to different conditions that have been studied by Sujana [17] and Kim et al. [18]. However, the drinking water plant-produced water supply sludge (WS) [19] in the coagulation process of producing water, whose structure and composition is different from those of alum sludge. Meanwhile, it has not been reported that using WS to remove fluoride from water and the adsorption characteristics of various conditions has not been studied. 

In this study, WS was used as an adsorbent for the removal of fluoride. Additionally, the effect of coexistence anion and various conditions, such as pH, dosage, and initial fluoride concentration, was investigated using the response surface methodology (RSM). This work can provide a new orientation for separating fluoride ions from water and offer a positive reference for solid waste recovery.

## 2. Materials and Methods

### 2.1. Samples Collection and Treatment

WS was taken from the Qujiang water supply plant (Xi’an, China) and air-dried in a clean yard, then crushed and sieved to the sizes (1 mm) and stored in airtight containers until further using. The original sludge sample was treated with following different methods: pyrolysis was accomplished at 873 K for 60 min in pyrolysis furnace [11], and the fuel oxidation sludge was yielded by firing at temperatures 873 K for 6 h in muffle furnace [20]. To get the hydrochloric acid acidified sludge, the untreated sludge was water-washed twice, then soaked in 5% hydrochloric acid for 24 h, and the supernatant was filtered off. The sludge was washed with deionized water for 7 to 8 times until it was neutral and was dried at a temperature of about 100 °C. The sulphuric acid acidified was the same process with 5% HCl acidified sludge, while the sulphuric acid was used instead of hydrochloric acid [21].

### 2.2. Experimental Process

Adsorption experiments were conducted by Organization for Economic Co-operation and Development (OECD). The adsorbent mixed with solution was placed in the flask with magnetic stirrer (Model 04803-02, Cole-Palmer-Instrument Company, Vernon Hills, IL, USA). The residual fluoride concentration was measured immediately after filtration using the microfiltration membrane. All the experiments were performed in triplicate, and the mean values were reported.

#### 2.2.1. Reagent and Standard Solutions

To simulate natural water, fluoride stock solution was prepared by dissolving anhydrous sodium fluoride (99.0%, NaF, Kemiou Chemical Reagent Co. Ltd., Tianjin, China) with distilled water. Standards F^−^ samples at a required concentration range were prepared using appropriate dilution of the stock solution.

The pH of the solution was adjusted by adding 0.1 mol/L HCl or NaOH solutions (Kemiou Chemical Reagent Co. Ltd., Tianjin, China). The pH values were periodically measured and readjusted until they were constant. The co-existing anions (SO_4_^2−^, NO_3_^−^, SiO_3_^2−^, PO_4_^−^,) solution was prepared with corresponding sodium salts to the concentration of 5–10 mg/L.

#### 2.2.2. Adsorption Kinetics and Isotherm

Adsorption experiment was carried out with four treated sludges at first. The fluoride removal efficiency of four treated sludges was investigated by using 2 g sludge and 48 mL of the 10 mg/L F^−^ stock solution in 200 mL conical flask, respectively, to keep the constant initial fluoride concentration of 2.4 mg/L.

In order to obtain an appropriate contact time between the HWS and fluoride ions, the samples were taken at time intervals of 2, 4, 8, 16, 20, 24, 30, 36, 40, 48, 60, and 70 min, then allowed to settle, and residual fluoride ion concentration was measured. The kinetics of fluoride adsorption on the HWS was determined under the initial fluoride of 2.4 mg/L and HWS dose at 12 g/L by using two different kinetic models, which are the pseudo-first-order model and pseudo-second-order model [22,23].

Isotherm experiments were conducted for the equilibration time of 70 mins by varying the fluoride concentration from 0.5 to 5 mg/L and the constant HWS dose at 15 g/L. The experimental data was fitted to Fruendlich and Langmuir isotherm models [24]. The removal efficiency (η) of fluoride ions and equilibrium sorption could be obtained by the Equations (1) and (2):

(1) $$\eta =\frac{C_{0}-C_{e}}{C_{0}}\times 100\%,$$

(2) $$Q_{e}=\frac{V\left(C_{0}-C_{e}\right)}{m},$$

where η (%) is the removal efficiency of fluoride ions, C_0_, C_e_ are the initial and equilibrium concentrations of fluoride ions (mg/L), Q_e_ is the equilibrium sorption (mg/g) at equilibrium, V (L) is the volume of the aqueous solution, and m is the mass (g) of adsorbent used in the experiments.

#### 2.2.3. Batch Experiments of Variable Condition

The main factors affecting the adsorption of HWS were studied by RSM, including sludge doses (5, 10, 15 g/L), initial fluoride concentration (1, 3, 5 mg/L), and pH (4, 7, 10). RSM is one such statistical technique and is used for designing experiments, building models, evaluating the effects of several variables, and obtaining the optimum conditions for responses with a limited number of planned experiments [25,26].

Initially, the main experiments were conducted according to Box–Behnken design (BBD) to facilitate the modeling and optimization of the process (*n* = 17). Batch experiments for modeling and optimization were conducted according to design matrix presented in Table 1. For each experiment, 100 mL of solution with desired fluoride concentrations and HWS dose was mixed at pH = 4, 7, 10, for 70 min, respectively. Finally, the supernatant was drained, and fluoride concentration was determined.

#### 2.2.4. Effect of Co-Existing Anions

The effect of co-existing anions (SO_4_^2−^, SiO_4_^4-^, PO_4_^3−^, NO_3_^−^) on fluoride adsorption efficiency of WS was also studied at constant fluoride concentration of 2.4 mg/L and adsorbent dose of 15 g/L. Anion (SO_4_^2−^, PO_4_^3−^, SiO_4_^4−^and NO_3_^−^) concentrations of 5, 10, 20, 30, and 50 mg/L were prepared by dissolving calculated amount of their sodium salt in 2.4 mg/L fluoride solution.

#### 2.2.5. The Examination of Removal Efficient from Water

To testify the removal efficiency of the fluoride ions from water under optimal conditions, the adsorption experiment was studied with three water samples. W1 was collected from the effluent of a typical fluorite tailing pond, and W2 and W3 were collected from the effluent of two different glass processing factories. The concentration of three water samples are shown in Table 2.

### 2.3. Analysis

An ionic-activity meter (PXS-215, Shanghai instrument electric science instrument, Shanghai, China) equipped with combination fluoride-selective electrode (PF-1, Shanghai instrument electric science instrument, Shanghai, China) was employed for the measurement of fluoride ion concentration. The pH was measured with pH meter (DELTA320, Mettler Toldedo, Zurich, Switzerland). The compositions of sludge were analyzed using X-ray diffraction (XRD, Shimadzu, Kyoto, Japan), and the surface morphologies of sludge were examined using scanning electron microscope (SEM, JSM-6700F, FEI NanoPorts, Hillsboro, AL, USA). The surface area, pore area, pore volume, and average pore size determinations were carried out by N_2_ adsorption isotherms using a Micrometrics ASAP 2020K surface area analyzer (Micromeritics Instrument Corp., Atlanta, GA, USA).

## 3. Results and Discussion

### 3.1. Effects of Four Treated Sludges when Removing Fluoride

**Figure 1:**
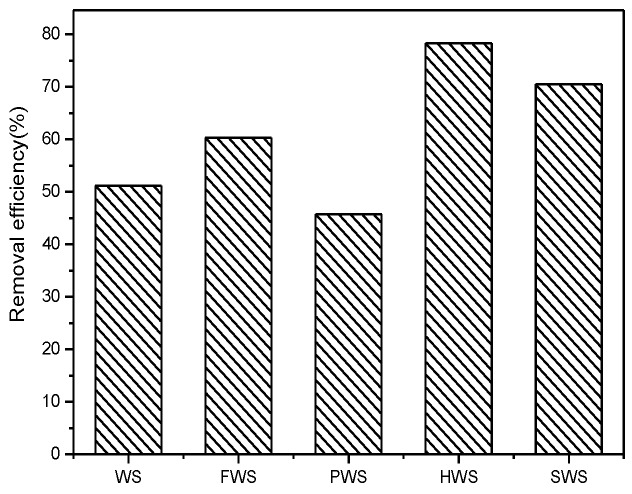
Removal efficiency of fluoride by different treated WS. Note: WS: water supply sludge, FWS: fuel oxidation-WS, PWS: pyrolysis WS, HWS: hydrochloric acid-WS, SWS: sulphuric acid-WS).

The compositions of WS for the experiment are shown in Table 3. The WS contained large number of Al_2_O_3_ and Fe_2_O_3_ through the coagulation with the addition of polyaluminum chloride (PAC), aluminum sulfate (Al_2_(SO_4_)_3_), and ferric chloride (FeCl_3_) in the process of producing water. SiO_2_ was brought by the sludge in the settlement process_._ Compared to the WS, alum sludge coming from the alum plant [17] contained almost the same content of Al_2_O_3_ and Fe_2_O_3_ but less SiO_2_ and more TiO_2_, which was decided by the raw materials and the production process. In order to improve the adsorption ability, the original sludge sample was treated with different methods. Meanwhile, fluorine removal experiments were carried out with the treated samples. The results shown in Figure 1 indicate that acid treatment can improve the ability of sludge to adsorb fluoride from water. Compared to other methods of treatment with original sludge, the sludge after acid treatment has the best efficiency, and HCl-acidified sludge is better than H_2_SO_4_. The fuel oxidation treatment can slightly improve the removal efficiency, while the pyrolysis displays a little suppression (Figure 1).

### 3.2. Equilibrium and Kinetics of Adsorption

**Figure 2:**
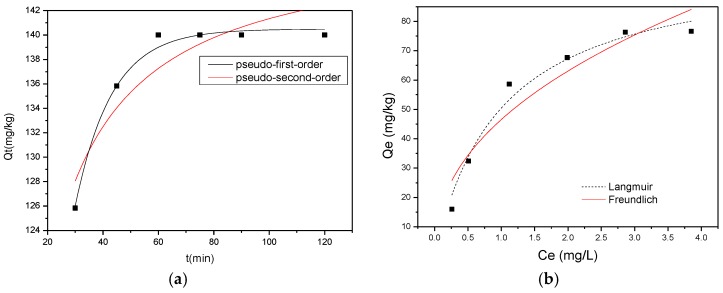
The fitted plot of (**a**) kinetics and (**b**) isotherm models.

Adsorption kinetics, demonstrating the solute uptake rate, is one of the most important characters that represent the adsorption efficiency of the HWS. Two possible models of kinetics were used to fit the experimental results (Figure 2a): (1) pseudo-first-order model and (2) pseudo-second-order model [27,28].

As shown in Table 4, the pseudo-first-order model has a better fit with the experimental data with the higher squared correlation coefficients (R^2^ = 0.9894). The adsorption reaction is fast during the initial 40 min, and equilibrium was reached around 70 min. This result was in agreement with the equilibrium time found for alum sludge and quartz, which are reported in other studies [17,24].

The analysis of Langmuir and Freundlich isotherm models [29] were presented in Figure 2b and Table 5. The R_L_ values in Langmuir model are favorable (0 < R_L_< 1) [30]. The higher regression correlation coefficient (0.9959) was observed for Langmuir model, indicating that the Langmuir model was the most suitable for describing the adsorption equilibrium, meaning the formation of fluoride ion at the outer surface of the HWS was monolayer coverage [22].

### 3.3. Adsorption Mechanism Analysis

#### 3.3.1. Surface Area Analysis

**Figure 3:**
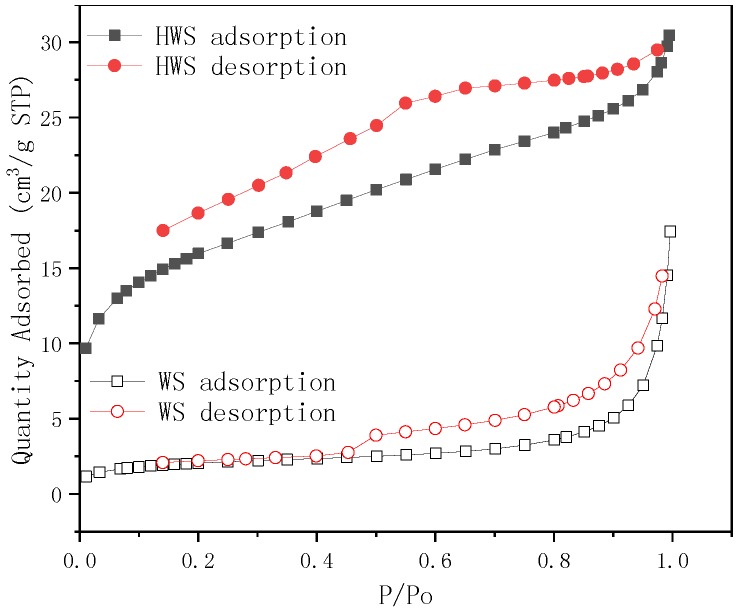
Adsorption-desorption isotherm. (STP: stardard tempreture (273.15K (0 °C) and pressure (100 kPa))*.*

To further explore the surface area of the untreated and hydrochloric acid water sludge samples, the BET adsorption experiment was employed. Adsorption-desorption N_2_ isotherms are shown in Figure 3, and the surface area and pore size data are listed in Table 6. N_2_ adsorption-desorption loops were not closed, because when the experiments were below 0.15 Pa pressure, irreversible adsorption occurred, and the adsorbed N_2_ could not be desorbed. This indicated that there was a strong adsorption potential in the micropores of the HWS, while the WS sample does not exist in this case, and Table 6 shows that the average pore size of HWS is 3.23694 nm, which is relatively smaller than the WS of 8.88669 nm, which also revealed the that there is no adsorption potential in the micropores of WS. In addition, the surface area and pore volume of HWS improved 6.82 and 7.93 times, respectively, compared to the untreated WS, so the adsorption capacity of HWS is higher than WS is reasonable.

#### 3.3.2. SEM and XRD Studies

**Figure 4:**
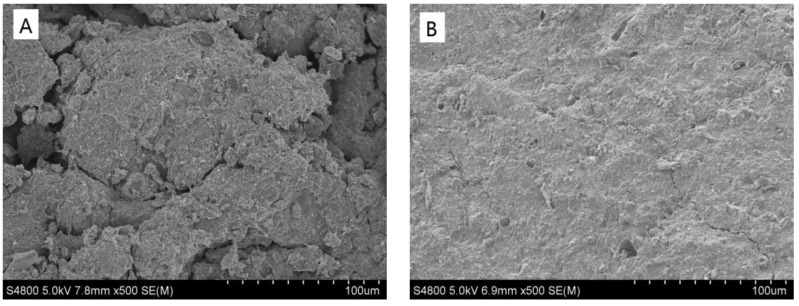
SEM of the adsorbent (**A**) before adsorption (**B**) after adsorption.

**Figure 5:**
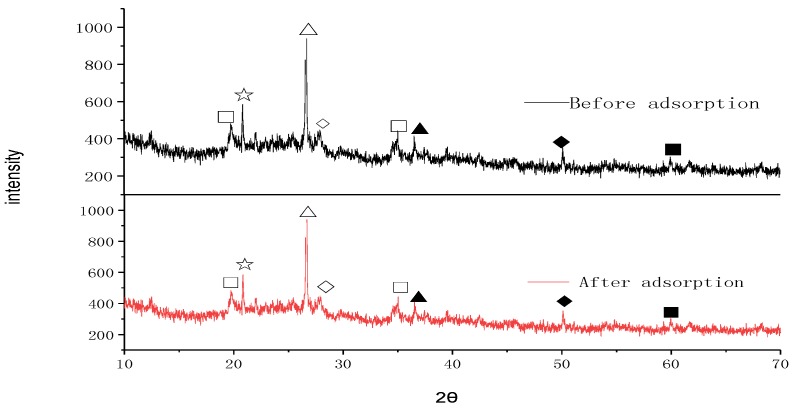
XRD spectra of HWS before and after the adsorption of fluoride, and the identified compounds: □ K_1.2_Al_4_Si_8_O_2_(OH)_2_·4H_2_O, ☆ TiFeCl_3_, △ SiO_2_, ◇ Al_2_SiO_5_, ▲ (Mg,Fe)_2_SiO_4_, ◆ Fe_0.4_Mg_0.76_SiO_3_, and ■ (Mg,Al,Fe)_6_(Si,Al)_4_O_10_(OH)_8_.

The SEM images of HWS before and after absorption are shown in Figure 4. There is no evident change observed after the adsorption. The XRD results (Figure 5) also revealed that only peaks intensity changed, while the diffractograms are similar, and no important changes in the structure of the adsorbent after adsorption are observed, indicating that the process was mainly physical adsorption-dominated. The identified compounds were K_1.2_Al_4_Si_8_O_2_(OH)_2_·4H_2_O, TiFeCl_3_, SiO_2_, Al_2_SiO_5_, (Mg,Fe)_2_SiO_4_, Fe_0.4_Mg_0.76_SiO_3_, and (Mg,Al,Fe)_6_(Si,Al)_4_O_10_(OH)_8_.

### 3.4. Effects of Variable Conditions on the Adsorption of Fluoride

The effects of variable conditions on fluoride removal were studied using RSM. As mentioned earlier, RSM based on BBD was employed to investigate the effects of three independent variables, HWS dose, pH, and initial concentration on the adsorption of fluoride by HWS. The BBD factorial design along with five replicates at central points is presented in Table 1. Design Expert 8.0.6 software (Stat-Ease Corporation, Minneapolis, MN, USA) was used for experimental design and analysis. Experimental data were fitted to a second-order polynomial model [31]:

(3) $$Y=b_{0}+\sum_{i=1}^{k}b_{i}x_{i}+\sum_{i=1}^{k}b_{\mathrm{ii}}x_{i}^{2}+\sum_{i=1}^{k-1}\sum_{j=i+1}^{k}b_{\mathrm{ij}}x_{i}x_{j}+\varepsilon ,$$

in which Y is the predicted response (Removal efficiency in %) used as dependent variable, x_i_ and x_j_ are the in dependent variables, b_0_ is the constant coefficient, b_i_ is the coefficient that determines the influence of variable i in the response, b_ij_ is the coefficient that determines the effect of interaction between variables i and j, b_ii_ is the parameter that determines the shape of the curve, and k is the number of variables studied [25,32]. 

RSM model and its validation based on the experimental results are presented in Table 2. Based on the experimental data, regression models using a second-order polynomial were represented by Equation (4), which was developed, after which statistically insignificant coefficients (*p*-value greater than 0.1) were excluded from the analysis.

(4) $$Y=7.794-5.965x_{1}+7.806x_{2}+7.825x_{3}+0.273x_{1}x_{3}-0.640{x\;}_{1}^{2}-0.635x_{2}^{2}-0.289x_{3\;}^{2\;}$$

The analysis of variance (ANOVA) for the proposed model and corresponding *p*-values and F-values for assessing the significance of the regression coefficients are presented in Table 7. A *p*-value of model less than 0.05 implies that the proposed model well predicts the experimental results at 5% confidence interval [21]. A large *p*-value for lack of fit (>0.05) is preferred, as it measures the model failure in representing data points in the experimental domain [33]. In this case, the *p*-value of lack of fit is 0.1792, implying that lack of fit of the model is insignificant. Adequate precision (AP) is the ratio of the predicted responses from the design points to their average standard deviation, which, for a good model fit, its desired value is 4 or more [21]. The ratio of 48.388 implies that the model is acceptable. The overall prediction performance of the model is described by coefficient of determination (R^2^). A high R^2^ value, close to 1, is desirable to ensure a satisfactory adjustment of the model to the experimental data [26]. The value of R^2^ = 0.9957 and a reasonable agreement with R^2^_adjusted_ is necessary [25,34]. In the present models, the values of R^2^_adjusted_ = 0.9901 was close to R^2^, indicating high significance of the model.

**Figure 6:**
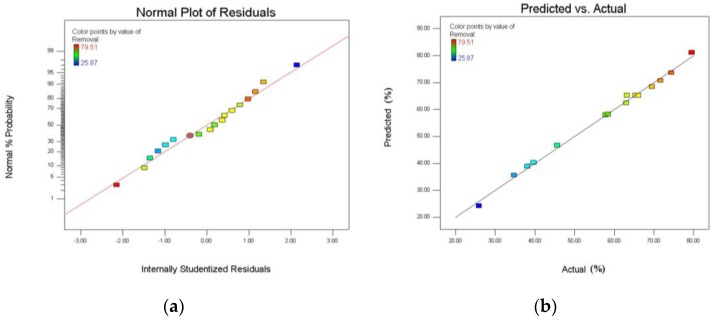
Assess the adequacy of the model used the plot of the residuals (**a**) Normal probability of residuals and (**b**) Predicted versus actual values.

A plot of the residuals was also used to assess the adequacy of the model. The residual plots of the models are presented in Figure 6a. The residuals are normally distributed if the points on the plot follow a straight line [31]. As Figure 6a illustrates, the assumption of normality is satisfied for the models [26]. Figure 6b presents the observed and predicted values. The statistical significances of the models are evident from Figure 6a, as observed and predicted values fit each other well. The statistical model can be used to predict the removal efficiency in this experiment in the range above.

**Figure 7:**
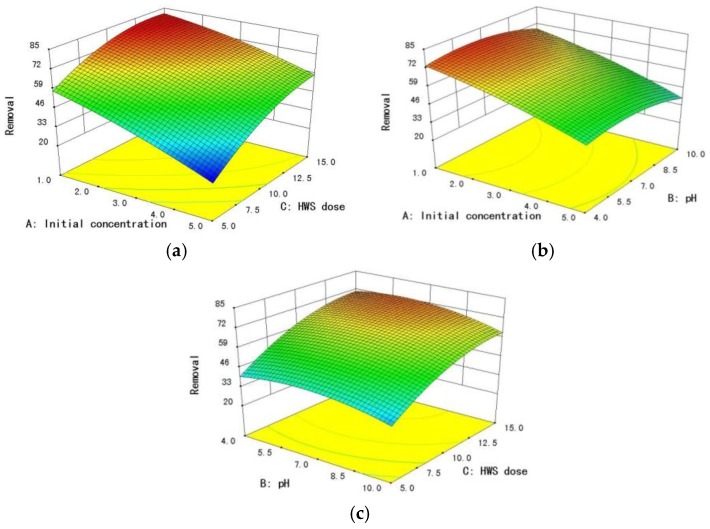
Response surface graphs: (**a**) response surface plot for fluoride removal as a function of HWS dose and initial concentration, (**b**) response surface plot for fluoride removal as a function of pH and initial concentration, and (**c**) response surface plot for fluoride removal as a function of HWS dose and pH.

Response surface graphs presented in Figure 7 demonstrate the effects of variables and their interactive effects on the removal of fluoride. These plots are generated as a function of two variables at the same time, keeping the third variable at a centre level. As shown in (a) and (b), the increase of initial fluoride concentration leads to the decrease of final efficiency. As shown in (c), the removal efficiency increased as the HWS dose increased under the same pH. The increase in fluoride adsorption was possibly attributed to the increase in availability of F^−^due to the presence of a greater number of active sites [35,36]. Considering initial concentration = 3 mg·L^−1^, the WHO standard for permissible limit of fluoride in water (≤1.5 mg·L^−1^), which would be fulfilled with HWS (≥6.17 g·L^−1^) in the neutral condition of the present work，can be less at pH = 6. However, the effect of removal efficiency of fluoride was more prominent by initial concentration compared to HWS dose and pH from (a) and (b), meaning initial concentration of fluoride has an adverse effect on its removal. Samarghandi reported a similar result in adsorption of fluoride [31]. At low initial concentration, most of fluoride will interact with the binding sites of the adsorbent, resulting in higher removal percentage. On the other hand, at high initial concentration, only some of the ions will combine with the finite available sites for binding [37].

Usually, pH has been seen as an important factor influencing adsorption of the crystalline form to the adsorbent. It has been reported that, in case of zeolite and activated alumina, the pH of zero charge (pHpzc) may vary from 5.5 to about 8.3 [24], and the optimum pH for maximum adsorption is between 5 and 7 [31,38]. The influence of the initial pH on the removal efficiency of this study is shown in Figure 7b,c. The percentage of fluoride removal remains nearly constant within the pH range of 4–7. Further increase in the pH of the solution slightly decreases the removal efficiency. The fluoride uptake capacity of this media is not affected in the pH range less than or equal to 7, possibly due to the presence of positively charged and neutral sites at the surface of the adsorbent [24]. The decline at pH > 7 may be due to the competition between OH^−^ and F^−^ [39]. This is in agreement with fluoride removal studies on activated alumina by other researchers. 

The numerical simulation optimum conditions for removal efficiency of fluoride using HWS were carried by RSM with the help of the desirability function. In this study, the desirability function approach was employed for optimization using Design Expert, which provides several possible options including minimum, maximum, target, within the range, none (only for response), and equal to (factors only) for choosing a desired goal for each variable and response [39]. The average values of confirmation tests in triplicates and predicted by the model under optimum conditions are presented in Table 7. As shown in Table 8, the removal efficiency of confirmation tests was closed to the prediction. The optimum removal efficiency of fluoride can reach 81.153% under the optimum condition: HWS dose of 14.10 g/L and pH value at 6.12. Meanwhile, the lower initial fluoride concentration is better.

### 3.5. Effect of Co-Existing Anions

**Figure 8:**
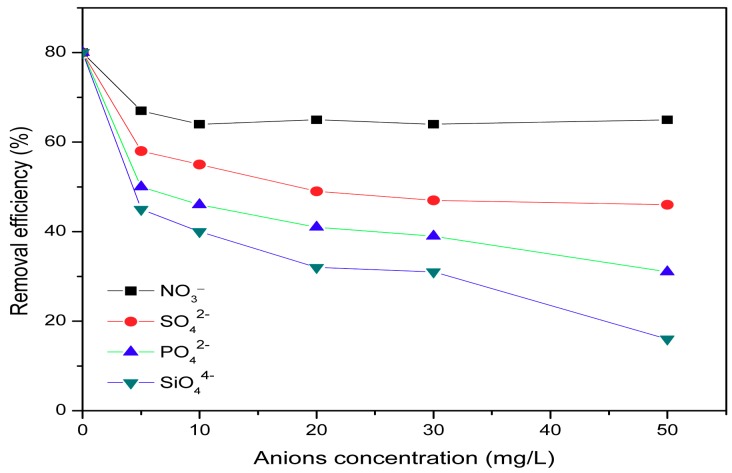
Effect of co-ions on adsorption.

As we all know, water contains other anions such as sulfate, phosphate, nitrate, and silicate in addition to fluoride. The results for the removal efficiency of HWS with concentration of each anion are shown in Figure 8. Some studies showed that the presence of other co-existing ions in water had an effect on fluoride removal [17,24,40,41]. It was observed that SO_4_^2−^, PO_4_^3−^, SiO_4_^4−^, and NO^3−^ ions showed a negative effect on removal of fluoride. The fluoride adsorption efficiency of the adsorbent decreased from about 80% to 14% in case of silicate and 29% in phosphate, and although the sulfate and nitrate showed slight influence on fluoride removal, the removal efficiency still decreased from 80% to 65% for nitrate and 46% for sulfate. This can be due to the competition between the anions and fluoride [41]. The competition ability of four anions followed the order SiO_4_^4−^ > PO_4_^3−^ > SO_4_^2−^ > NO_3_^−^; the results are in good agreement with similar work done by others [24] for activated alumina.

### 3.6. The Adsorption Efficiency from Water

The adsorption experiment results with wastewater samples are shown in Table 9. To examine the optimal removal efficiency, the 14.1g/L of adsorbent was first added. Results showed that the removal efficiencies were 79.21% for W1, 79.84% for W2, and 80.67% for W3, respectively, which was in agreement with the results obtained from RSM analysis, i.e., the removal efficiency for the lower initial fluoride concentration is better, for example, and the fluoride concentration of W3 was 8.6 mg/L, relating to a highest removal efficiency of 80.67%. However, once the optimal removal efficiency was acquired with such high adsorbent dose, the adsorption capacity inevitably decreased and was significantly lower than the adsorbents of other studies. In order to make the results of this study comparable to the others, the adsorbent dose was drastically reduced to 2 g/L to acquire a satisfy adsorption capacity. As a result, the harvested maximum adsorption capacity was 2.03 mg/g appearing at adsorption processes in W1 sample, which is lower than composite adsorbent, desugared reed root, and lignite from other literatures, while higher than pumice, modified montmorillonite, modified hematite, and modified zeolite (Table 10). Of course, its greatly decreased removal efficiency (only 20.10%) was foreseeable.

## 4. Conclusions

This work has shown that WS can be considered as a promising material for removing fluoride from poor quality water. The conclusions drawn from this study are given below:

(1) Acid treatment and high temperature ranges found as the conditions for better fluoride sorption and hydrochloric acid treatment will gain the best efficiency for removing fluoride for WS.
(2) A model of adsorption has been proposed for the adsorption of F^−^ onto HWS. The results gained from this study were well described by the theoretical Langmuir isotherms. The values of the equilibrium parameter and RL indicated that the F^−^/treated HWS system was favorable. Kinetic studies reveal that the adsorption is first order. Thermodynamic parameters were calculated, indicating that the adsorption was spontaneous.
(3) RSM based on BBD was employed to investigate the effects of the three independent variables, namely, HWS dose, pH, and initial concentration on the adsorption of fluoride by HWS. The optimum removal efficiency of fluoride from wastewater using HWS was observed with the help of RSM, and it can reach 81.153% under the optimum condition: HWS dose of 14.10 g/L, at pH = 6.12.
(4) In addition, the results showed that presence of other co-exiting ions, such as SO_4_^2−^, PO_4_^3−^, SiO_4_^4−^, and NO_3_^−^ anions show a negative effect on the removal of fluoride to varying degrees, in the order SiO_4_^4−^ > PO_4_^3−^ > SO_4_^2−^ > NO_3_^−^.

## Figures and Tables

**Table 1:** BBD matrix and its observed and predicted responses.

Run	*x_1_*-Initial Concentration (mg/L)	*x_2_*-pH	*x_3_*-HWS dose (g/L)	Removal Efficiency (%)		ε
				Y_exp_	Y_pre_	
1	3	10	5	34.72	35.61	−0.89
2	3	4	15	71.62	70.73	0.89
3	3	7	10	63.17	65.20	−2.03
4	1	7	5	57.84	57.98	−0.14
5	3	10	15	63.03	62.42	0.61
6	5	4	10	45.65	46.68	−1.03
7	3	7	10	65.32	65.20	0.12
8	3	7	10	65.71	65.20	0.51
9	5	7	5	25.87	24.23	1.64
10	1	4	10	74.42	73.67	0.75
11	3	7	10	65.78	65.20	0.58
12	3	7	10	66.03	65.20	0.83
13	1	10	10	69.53	68.49	1.04
14	1	7	15	79.51	81.15	−1.64
15	5	10	10	38.12	38.87	−0.75
16	3	4	5	39.68	40.29	−0.61
17	5	7	15	58.45	58.31	0.14

Note: HWS: hydrochloric acid treated sludge; bbd: Box–Behnken design. Y_exp_: the experimental obtained removal efficiency; Y_pre_: the predicted removal efficiency used BBD modeling. BBD: Box–Behnken design.

**Table 2:** The water quality of three water samples.

Samples	F^−^ (mg/L)	SS (mg/L)	pH
W1	20.2	615.0	9.10
W2	15.3	45.0	7.40
W3	8.6	34.0	7.42

Note: SS: suspended solid.

**Table 3:** Composition of the chemical constituents in WS.

Chemical Composition	WS (%)	Alum Sludge(%) [17]
Al_2_O_3_	47.56	47.20
SiO_2_	28.54	1.60
Fe_2_O_3_	4.30	7.18
TiO_2_	1.47	20.65
Cl^−^	2.14	-
LOI	14.32	19.00

Note: WS: water supply sludge.

**Table 4:** Kinetic parameters and statistical parameters of the two kinetic models.

Adsorbent	Q_e,exp_ (mg/g)	Pseudo-First Order			Pseudo-Second Order		
		R^2^	K_1_ (1/h)	Q_e,cal_ (mg/g)	R^2^	K_2_ (kg/mg·h)	Q_e,cal_ (mg/g)
HWS	0.1402	0.9894	0.0757	0.1405	0.8286	1.5	0.1479

Note: Q_e,exp_ refers to actual equilibrium adsorption capacity; Q_e,cal_ refers to the fitted theoretical equilibrium adsorption capacity; K_1_, K_2_ is the rate constant of the kinetics models.

**Table 5:** Adsorption isotherm constants for fluoride adsorption onto HWS.

Langmuir				Freundlich		
Q_m_ (mg/g)	b (L/mg)	R^2^	R_L_	K_F_ (mg/g)·(L/mg)^1/n^	1/n	R^2^
0.24643	1.8547	0.9959	0.7294	0.1455	0.4165	0.9537

**Table 6:** The surface area, pore volume, and pore size of the WS and HWS.

Samples	Surface Area (m^2^/g)	Pore Volume (m^3^/g)	Average Pore Size (nm)
WS	6.85680	0.000723	8.88669
HWS	53.59230	0.006453	3.23694

**Table 7:** Analysis of variance (ANOVA) test for removal (%) of fluoride.

Source	Sum of Squares	df	Mean Square	F-value	*p*-value *p* > F	Significance
Model	3782.08	9	420.23	178.60	<0.0001	significant
A-initial concentration	1602.06	1	1602.06	680.88	<0.0001	
B-pH	84.31	1	84.31	35.83	0.0006	
C-HWS dose	1638.78	1	1638.78	696.48	<0.0001	
AB	1.74	1	1.74	0.74	0.4180	
AC	29.76	1	29.76	12.65	0.0093	
BC	3.29	1	3.29	1.40	0.2753	
A^2^	27.56	1	27.56	11.71	0.0111	
B^2^	137.45	1	137.45	58.42	0.0001	
C^2^	219.85	1	219.85	93.44	<0.0001	
Residual	16.47	7	2.35			
Lack of Fit	11.05	3	3.68	2.72	0.1792	not significant
Pure Error	5.42	4	1.36			
Cor Total	3798.55	16				

R^2^ = 0.9957, R^2^_adjusted_ = 0.9901, R^2^_predicted_ = 0.9512, adequate precision = 48.388. A, B, C refer to the linear entries (initial concentration, pH, HWS dose, respectively) of the analysis of variance and A^2^, B^2^, C^2^ are its quadric entries; AB, AC, BC refer to the interaction item (initial concentration–pH, initial concentration–HWS dose, pH–HWS dose, respectively, df refers to the degree of freedom).

**Table 8:** Optimum conditions for fluoride removal.

Item	Initial Concentration (mg/L)	pH	HWS Dose (g/L)	Removal (%)	Desirability
Predicted	linear	6.12	14.10	81.921	0.756
Observed	linear	6.12	14.10	81.153	-

**Table 9:** The adsorption capacity of HWS for three water samples with different adsorbent doses.

Adsorbent	Adsorbent Dose (g/L)	Initial Concentration (mg/L)	Adsorption Capacity (mg/g)	Removal Efficiency (%)
HWS	14.10	2.40	0.141	82.84
	2.00	2.40	0.625	52.08
	14.10	20.20 (W1)	1.135	79.23
	2.00	20.00 (W1)	2.030	20.10
	14.10	15.30 (W2)	0.866	79.81
	2.00	15.30 (W2)	1.940	25.36
	14.10	8.60 (W3)	0.492	80.67
	2.00	8.60 (W3)	1.535	35.70

**Table 10:** Comparison of properties between HWS adsorbent and other adsorbents.

Adsorbent	Adsorbent Dose (g/L)	Initial Concentration (mg/L)	Adsorption Capacity (mg/g)	Reference
Composite absorbent	1.5	5.24	3.07	[42]
Pumice	2.29	2.8	0.31	[43]
Desugared reed root	0.1	2.4	2.136	[44]
Lignite	3	90	6.9	[45]
Modified montmorillonite	1	10	0.696	[46]
Modified hematite	-	5.8	0.53	[47]
Modified zeolite	-	0.1	1.766	[38]
HWS	2.0	20.2 (W1)	2.03	This study

## References

[1] Ozsvath D.L. (2008). Fluoride and environmental health: A review. Rev. Environ. Sci. Biol..

[2] Velazquez-Jimenez L.H., Hurt R.H., Matos J., Rangel-Mendez J.R. (2014). Zirconium-carbon hybrid sorbent for removal of fluoride from water: Oxalic acid mediated Zr(IV) assembly and adsorption mechanism. Environ. Sci. Technol..

[3] Mohapatra M., Anand S., Mishra B.K., Giles D.E., Singh P. (2009). Review of fluoride removal from drinking water. J. Environ. Manag..

[4] Aoudj S., Drouiche N., Hecini M., Ouslimane T., Palaouane B. (2012). Coagulation as a Post-Treatment Method for the Defluoridation of Photovoltaic Cell Manufacturing Wastewater. Procedia Eng..

[5] Tor A. (2007). Removal of fluoride from water using anion-exchange membrane under Donnan dialysis condition. J. Hazard. Mater..

[6] Ho L.N., Ishihara T., Ueshima S., Nishiguchi H., Takita Y. (2004). Removal of fluoride from water through ion exchange by mesoporous Ti oxohydroxide. J. Colloid Interface Sci..

[7] Tripathy S.S., Bersillon J.L., Gopal K. (2006). Removal of fluoride from drinking water by adsorption onto alum-impregnated activated alumina. Sep. Purif. Technol..

[8] Yu X.L., Tong S.R., Ge M.F., Zuo J.C. (2013). Removal of fluoride from drinking water by cellulose @ hydroxyapatite nanocomposites. Carbohyd. Polym..

[9] Pan B.C., Xu J.S., Wu B., Li Z.G., Liu X.T. (2013). Enhanced removal of fluoride by polystyrene anion exchanger supported hydrous zirconium oxide nanoparticles. Environ. Sci. Technol..

[10] Fan X., Parker D.J., Smith M.D. (2003). Adsorption kinetics of fluoride on low cost materials. Water Res..

[11] Márquez-Mendoza S., Jiménez-Reyes M., Solache-Ríos M., Gutiérrez-Segura E. (2012). Fluoride removal from aqueous solutions by a carbonaceous material from pyrolysis of sewage sludge. Water Air Soil Pollut..

[12] Ghorai S., Pant K.K. (2005). Equilibrium, kinetics and breakthrough studies for adsorption of fluoride on activated alumina. Sep. Purif. Technol..

[13] Xu Y.M., Ning A.R., Zhao J. (2001). Preparation and defluorination performance of activated cerium(IV) oxidesimcm-41 adsorbent in water. J. Colloid Interface Sci..

[14] Wajima T., Umeta Y., Narita S., Sugawara K. (2009). Adsorption behavior of fluorideions using a titanium hydroxide-derived adsorbent. Desalination.

[15] Turner B.D., Binning P., Stipp S.L.S. (2005). Fluoride removal by calcite: Evidence for fluorite precipitation and surface adsorption. Environ. Sci. Technol..

[16] Hu Y.S., Zhao Y.Q., Zhao X.H., Jeyakumar L. (2012). High Rate Nitrogen Removal in an Alum Sludge-Based Intermittent Aeration Constructed Wetland. Environ. Sci. Technol..

[17] Sujana M.G., Thakur R.S., Rao S.B. (1998). Removal of Fluoride from aqueous solution by using alum sludge. J. Colloid Interface Sci..

[18] Kim Y.S., Kim D.H., Yang J.S., Baek K. (2012). Adsorption Characteristics of As(III) and As(V) on Alum Sludge from Water Purification Facilities. Sep. Sci. Technol..

[19] Razali M., Zhao Y.Q., Bruen M. (2007). Effectiveness of a drinking-water treatment sludge in removing different phosphorus species from aqueous solution. Sep. Purif. Technol..

[20] Wajima T., Rakovan J.F. (2013). Removal of fluoride ions using calcined paper sludge. J. Therm. Anal. Calorim..

[21] Nair A.T., Ahammed M.M. (2013). The reuse of water treatment sludge as a coagulant for post-treatment of UASB reactor treating urban wastewater. J. Clean. Prod..

[22] Wahab M.A., Jellali S., Jedidi N. (2010). Ammonium biosorption onto sawdust: FTIR analysis, kinetics and adsorption isotherms modeling. Bioresour. Technol..

[23] Lv L., He J., Wei M., Evans D.G., Zhou Z. (2007). Treatment of high fluoride concentration water by MgAl-CO_3_ layered double hydroxides: Kinetic and equilibrium studies. Water Res..

[24] Nigussie W., Zewge F., Chandravanshi B.S. (2007). Removal of excess fluoride from water using waste residue from alum manufacturing process. J. Hazard. Mater..

[25] Zhang H., Li Y., Wu X. (2012). Statistical experiment design approach for the treatment of landfill leachate by photoelectro-Fenton process. J. Environ. Eng..

[26] Nair A.T., Makwana A.R., Ahammed M.M. (2014). The use of response surface methodology for modelling and analysis of water and wastewater treatment processes: A review. Environ. Sci. Technol..

[27] Cheng D.H., Yang S.K., Zhao Y., Chen J. (2013). Adsorption behaviors of Oxytetracycline onto sediment in the Weihe River, Shaanxi, China. J. Chem..

[28] Zhao Y., Yang S.K., Wang G., Han M. (2015). Adsorption behaviors of Acetaminophen onto the colloid in sediment. Pol. J. Environ. Stud..

[29] Cengeloĝlu Y., Kir E., ErsÖz M. (2006). Removal of fluoride from aqueous solution by using red mud. Sep. Purif. Technol..

[30] Gopal V., Elango K.P. (2007). Equilibrium, kinetic and thermodynamic studies of adsorption of fluoride onto plaster of Paris. J. Hazard. Mater..

[31] Samarghandi M.R., Khiadani M., Foroughi M., Nasab H.Z. (2016). Defluoridation of water using activated alumina in presence of natural organic matter via response surface methodology. Environ. Sci. Pollut. R..

[32] Bashir M.J., Isa M.H., Kutty S.R.M., Awang Z.B., Aziz H.A., Mohajeri S. (2009). Landfill leachate treatment by electrochemical oxidation. Waste Manag..

[33] Mohajeri S., Aziz H.A., Isa M.H., Zahed M.A., Adlan M.N. (2010). Statistical optimization of process parameters for landfill leachate treatment using electro-Fenton technique. J. Hazard. Mater..

[34] Moghaddam S.S., Moghaddam M.R.A., Arami M. (2010). Coagulation/flocculation process for dye removal using sludge from water treatment plant: Optimization through response surface methodology. J. Hazard. Mater..

[35] Sundaram C.S., Viswanathan N., Meenakshi S. (2008). Defluoridation chemistry of synthetic hydroxyapatite at nano scale: Equilibrium and kinetic studies. J. Hazard. Mater..

[36] Gao S., Cui J., Wei Z. (2009). Study on the fluoride sorption of various apatite materials in aqueous solution. J. Fluor. Chem..

[37] Kumar E., Bhatnagar A., Kumar U., Sillanpää M. (2011). Defluoridation from aqueous solutions by nano-alumina: Characterization and sorption studies. J. Hazard. Mater..

[38] Zhang Z.J., Tan Y., Zhong M.F. (2011). Defluorination of wastewater by calcium chloride modified natural zeolite. Desalination.

[39] Mourabet M., Rhilassi A.E., Boujaady H.E., Bennani-Ziatni M., Hamri R.E., Taitai A. (2012). Removal of fluoride from aqueous solution by adsorption on apatitic tricalcium phosphate using box–behnken design and desirability function. Appl. Surf. Sci..

[40] Kamble S.P., Jagtap S., Labhsetwar N.K., Thakare D., Godfrey S., Devotta S., Rayalu S.S. (2007). Defluoridation of drinking water using chitin, chitosan and lanthanum-modified chitosan. Chem. Eng. Sci..

[41] Vinitnantharat S., Kositchaiyong S., Chiarakorn S. (2010). Removal of fluoride in aqueous solution by adsorption on acid activated water treatment sludge. Appl. Surf. Sci..

[42] Chen J.X., Chen H.H., Li X.H., He X.J. (2017). Preparation and application of zirconium-modified chitosan-zeolite-kaolin composite absorbent for fluoride removal from water. J. Environ. Health.

[43] Malakootian M., Moosazadeh M., Yousefi N., Fatehizadeh A. (2011). Fluoride removal from aqueous solution by pumice: Case study on Kuhbonan water. Afr. J. Environ. Sci. Technol..

[44] Song R., Yang S.K., Xu H.Y., Wang Z.Z., Chen Y.Y., Wang Y.Y. (2018). Adsorption Behavior and Mechanism for the Uptake of Fluoride Ions by Reed Residues. Int. J. Environ. Res. Public Health.

[45] Zhou C.Q., Deng X.H., Liu H.M., Li Z.W. (2006). Treatment of aqueous solution containing fluoride by absorption process. Technol. Water Treat..

[46] Bia G., de Pauli C.P., Borgnino L. (2012). The role of Fe(III)modified montmorillonite on fluoride mobility: Adsorptionexperiments and competition with phosphate. J. Environ. Manag..

[47] Teutli-Sequeira A., Martı’nez-Miranda V., Solache-Rı’os M., Linares-Herna’ndez I. (2013). Aluminum and lanthanum effects in natural materials on the adsorption of fluoride ions. J. Fluor. Chem..

